# In Silico Detection of Sequence Variations Modifying Transcriptional Regulation

**DOI:** 10.1371/journal.pcbi.0040005

**Published:** 2008-01-18

**Authors:** Malin C Andersen, Pär G Engström, Stuart Lithwick, David Arenillas, Per Eriksson, Boris Lenhard, Wyeth W Wasserman, Jacob Odeberg

**Affiliations:** 1 Department of Gene Technology, School of Biotechnology, AlbaNova University Center, Royal Institute of Technology (KTH), Stockholm, Sweden; 2 Atherosclerosis Research Unit, Department of Medicine, Center for Molecular Medicine, Karolinska University Hospital—Solna, Stockholm, Sweden; 3 Computational Biology Unit—Bergen Center for Computational Science and Sars Centre for Marine Molecular Biology, University of Bergen, HIB, Bergen, Norway; 4 Program for Genomics and Bioinformatics, Department of Cell and Molecular Biology, Karolinska Institutet, Stockholm, Sweden; 5 Ontario Cancer Institute, Princess Margaret Hospital, University of Toronto, Toronto, Ontario, Canada; 6 Centre for Molecular Medicine and Therapeutics (CMMT), Child and Family Research Institute, Department of Medical Genetics, University of British Columbia, Vancouver, British Columbia, Canada; 7 Karolinska Biomics Center, Karolinska University Hospital, Stockholm, Sweden; Washington University, United States of America

## Abstract

Identification of functional genetic variation associated with increased susceptibility to complex diseases can elucidate genes and underlying biochemical mechanisms linked to disease onset and progression. For genes linked to genetic diseases, most identified causal mutations alter an encoded protein sequence. Technological advances for measuring RNA abundance suggest that a significant number of undiscovered causal mutations may alter the regulation of gene transcription. However, it remains a challenge to separate causal genetic variations from linked neutral variations. Here we present an in silico driven approach to identify possible genetic variation in regulatory sequences. The approach combines phylogenetic footprinting and transcription factor binding site prediction to identify variation in candidate cis-regulatory elements. The bioinformatics approach has been tested on a set of SNPs that are reported to have a regulatory function, as well as background SNPs. In the absence of additional information about an analyzed gene, the poor specificity of binding site prediction is prohibitive to its application. However, when additional data is available that can give guidance on which transcription factor is involved in the regulation of the gene, the in silico binding site prediction improves the selection of candidate regulatory polymorphisms for further analyses. The bioinformatics software generated for the analysis has been implemented as a Web-based application system entitled RAVEN (regulatory analysis of variation in enhancers). The RAVEN system is available at http://www.cisreg.ca for all researchers interested in the detection and characterization of regulatory sequence variation***.***

## Author Summary

DNA sequence variations (polymorphisms) that affect the expression levels of genes play important roles in the pathogenesis of many complex diseases. Compared with genetic variations that alter the amino acid sequences of encoded proteins, which are relatively easy to identify, sequence variants that affect the regulation of genes are difficult to pinpoint among the large amount of nonfunctional polymorphisms located in the vicinity of genes. Computational methods to distinguish functional from neutral variations could therefore prove useful to direct limited laboratory resources to sites most likely to exhibit a phenotypic effect. In this paper we present a Web-based tool for the identification of genetic variation in potential transcription factor binding sites. This tool can be used by any scientist interested in the characterization of regulatory polymorphisms. Using experimentally verified regulatory polymorphisms and background data collected from the literature, we evaluate the method's capacity to identify regulatory genetic variation, and we discuss the limitations of its application.

## Introduction

Medical genetics research has produced a continuous stream of successes in which functional defects in genes have been linked to disease phenotypes. Such advances can lead to improved diagnostics, therapies, and therapeutics. While most discovered genetic defects create missense or nonsense substitutions in protein-coding sequences, there remain disease-associated genes for which there is no difference in protein-coding information between individuals of different phenotypes. Examples of the latter include CD36 type II deficiency [1], phenotypic variances in plasma Dopamine-beta-hydroxylase [2], and serum angiotensin I converting enzyme levels [3]. Since there is no change in the encoded proteins, some of these genes may be aberrantly expressed as a result of genetic changes in key regulatory sequences affecting the binding of transcription factors and thereby the rate of transcription.

There are few confirmed examples of regulatory genetic variants with a pronounced impact on disease. A common TA-repeat polymorphism in the cis-regulatory TATA box of the *UGT1A1* gene causes Gilbert syndrome in homozygous humans [4]. The variation has pharmacokinetic consequences: the reduced expression of the protein leads to altered glucuronidation of several drugs. Another example is a noncoding sequence variant of the *α-globin* gene cluster, which has been shown to generate a new transcriptional promoter by creating a novel functional binding site for GATA-1 [5]. The mutation was detected in Melanesian patients with a variant type of the inherited blood disorder α-thalassemia, in which patients have reduced expression of *α-globin* genes and anemia. Other examples of regulatory genetic variations include two mutations in HNF-1 binding sites in the promoter of the alpha fetoprotein, causing hereditary persistence of the protein which is otherwise only expressed in the fetus [6]. Usually, however, the effect of genetic variation on gene transcription is less pronounced, or merely associated with increased disease risk [7–11]. Several groups have compiled lists of regulatory sequence variations associated with allele-specific expression patterns [12–14].

Large-scale genomics studies have demonstrated that allele-specific differences in gene expression are common (reviewed in [15]), and that a portion of the differences can be attributed to genetic variation in noncoding regulatory regions [16–18]. The high frequency of observed allelic expression differences in mice [19] has been supported by similar rates observed with human samples [20,21]. Genome-wide mappings of expression levels in model organisms have revealed significantly higher fractions of polymorphisms close to genes for which altered mRNA expression levels between individuals have been linked to the locus of the same gene (self-linkage) compared to genes with no self-linkage [22,23]. While numerous studies have identified genes with allelic differences in regulation, it is still a challenge to separate causal genetic variations from linked neutral variations.

Computational methods to distinguish functional variations from neutral variations could prove useful to direct limited laboratory resources to sites most likely to exhibit a phenotypic effect. The identification of potential transcription factor binding sites (TFBSs) in DNA is fundamental to regulatory analysis. A traditional bioinformatics approach to predict TFBSs is through the application of binding site profile models known as position-specific weight matrices (PWMs) [24]. Such matrix models assign a score to each candidate binding sequence. In many cases, the models accurately predict in vitro binding properties of the corresponding transcription factors [25]. However, the low specificity of these models precludes their use for the detection of in vivo biologically relevant sites in the absence of additional information [26]. One type of additional information that is readily available is the evolutionary conservation of functional noncoding genomic sequence. This is the underlying principle behind phylogenetic footprinting, which can be used to eliminate regions less likely to contain cis-acting regulatory sites and thereby increase the specificity of predictions generated with position-specific weight matrices [27–29]. Restricting the search for genetic variation to predicted TFBSs in conserved regions can be expected to increase the likelihood of identifying functional variants. Such predictions are interesting if the putative TFBS is consistent with the biology of the associated disease.

Here we present an in silico driven approach to selecting genetic variation likely to influence gene regulation. We combine phylogenetic footprinting with detection of effects of genetic variation on putative TFBSs to identify variation with the potential to alter gene regulation. We test our in silico approach on genes with documented regulatory genetic variation and compare the results to a large set of background SNPs, to evaluate the enrichment of regulatory SNPs by our selection method. We introduce RAVEN (regulatory analysis of variation in enhancers), a Web interface to our application, that enables the scientific community to apply our approach to their genes of interest.

## Results

### Defining the Differences in TFBS Scores between Alleles for Various Mutation Types

**Figure 1:**
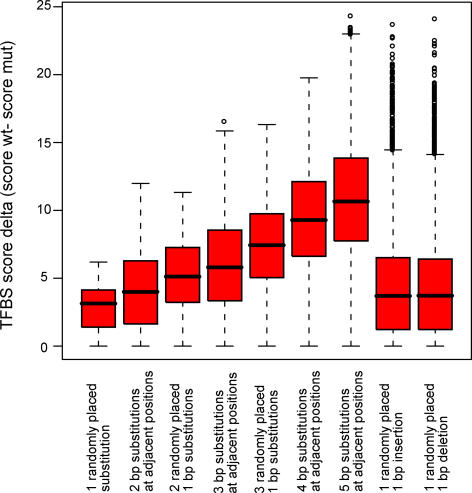
Impact on TFBS Score of Mutations Inserted into Synthesized TFBS Sequences The boxes correspond to score deltas for (from left to right) 1 bp substitutions, 2 bp substitutions at adjacent positions, two randomly placed 1 bp substitutions, 3 bp substitutions both in adjacent and at random positions, four randomly placed base pair substitutions, five randomly placed substitutions, one randomly placed 1 bp insertion, and one randomly placed 1 bp deletion.

Stormo and colleagues have shown that PWM representations of TFBSs gives scores that are proportional to the binding energy between the DNA sequence and the protein [30] (see Figure S1A and S1B for a demonstration of two examples where this holds true). This implies that matrix models of TFBSs can be used to estimate the effect on the transcription factor binding affinity of genetic variation in a regulatory region. Our in silico prediction tool identifies polymorphisms for which the assigned score to a TFBS model differs between the two allelic sequences. To define the expected ranges of allelic differences in TFBS scores for various types of mutations, we generated panels of simulated binding sites based on the distribution of bases at each position of the TFBS frequency matrices in the JASPAR database [31], representing wild-type TFBS sequences. We then inserted mutations into the generated sequences to produce a collection of synthetic regulatory sequence variants. Various types of mutations, including 1–5 bp substitutions, 1 bp insertions, and 1 bp deletions were introduced into the generated sequences; see Methods for a detailed description of the mutation classes. For every generated sequence, we computed a TFBS score delta by comparing the TFBS score for the wild-type allele (the sequence generated from the frequency matrix of the TFBS) with the one obtained for the mutated allele. For an explanation of the scoring system and its scale, see [24]. The box plot in Figure 1 shows that the majority of the simple 1 bp substitutions gives score deltas below four, and mutation of additional bases gives higher score deltas. Insertion and deletion polymorphisms behave similarly to each other, with approximately the same median value of the score deltas as for the 1 bp substitutions, but with a higher value of the third quartile and more outliers.

### Analysis of Differences in TFBS Scores between Alleles of Documented Regulatory and Background SNPs

**Figure 2:**
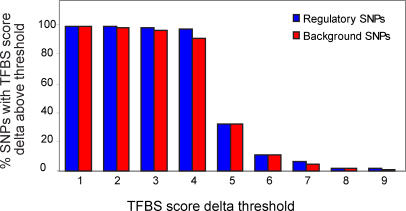
Fractions of Regulatory and Background SNPs Overlapping Predicted TFBSs SNPs were analyzed using all transcription factors in the JASPAR database, and using TFBS score delta thresholds between one and nine.

To assess the applicability of the approach to real sequences, we tested the PWM models of TFBSs on instances of genetic variation shown experimentally to affect the regulation of their corresponding genes. We compiled a list of 104 examples of experimentally verified regulatory 1 bp substitution polymorphisms. For comparison, we also compiled a list of 4,000 background 1 bp substitutions from dbSNP with a minor allele frequency exceeding 0.05. We tested all SNPs for overlap with the TFBS models in the JASPAR database, and recorded the score deltas that fulfilled the criteria described in Methods. We then calculated the fractions of SNPs that were retained for various TFBS score delta thresholds. Figure 2 shows the fractions of retained regulatory and background SNPs for the score delta thresholds of 1 unit increments between one and nine.

From Figure 2, it is apparent that the difference in the distributions of score deltas between the regulatory SNPs (rSNPs) and the background data is very small (median score deltas for the SNPs that overlap with a TFBS according to the search criteria are 4.8 and 4.9 for the regulatory and background SNPs respectively). When we restricted the search to TFBS models with a minimum specificity of ten bits (72 out of the 123 models in JASPAR fulfilled this more selective criterion), the fraction of retained SNPs decreased to approximately 75% for low TFBS score deltas. However, there was still no significant enrichment of rSNPs (unpublished data). These results indicate that the application of PWM models of TFBSs alone does not enrich for rSNPs.

### Phylogenetic Footprinting Enriches for Regulatory SNPs

**Figure 3:**
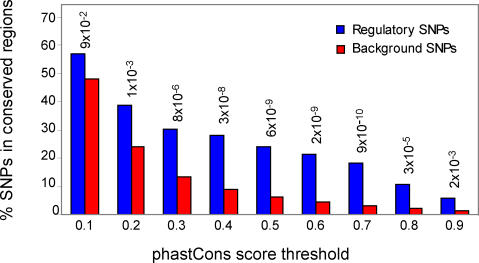
Fractions of Regulatory and Background SNPs in Evolutionary Conserved Regions SNPs were given the mean phastCons scores from multiple alignments of human, chimp, mouse, rat, dog, chicken, fugu, and zebrafish in windows of 21 bp centered at the SNPs. The fractions of SNPs located within conserved regions were calculated for mean phastCons score thresholds between 0.1 and 0.9. For every threshold a Fisher's exact test was performed to test if there was a significantly different frequency of successes in the regulatory versus the background SNP sets; *p*-values are indicated above each pair of bars.

We tested the application of phylogenetic footprinting to assess the method's capacity to enrich for bona fide rSNPs. The underlying principle behind phylogenetic footprinting is that functional noncoding elements are more likely to be evolutionarily conserved than nonfunctional surrounding sequence. Restricting the search for regulatory genetic variation to conserved regions is thus likely to increase the enrichment of functional sites. We therefore tested how often the upstream fraction of our experimentally verified regulatory polymorphisms was located within conserved genomic regions. Conservation of the rSNP positions was quantified using the phastCons scores [32] available at the UCSC genome browser from alignments between the May 2004 release of the human genome and chimp, mouse, rat, dog, chicken, fugu, and zebrafish. We performed similar testing for 26,044 background SNPs from dbSNP that are located within 10 kb upstream of human genes with known mouse orthologs. Figure 3 shows that the SNPs with documented effect on gene regulation are more frequently located within evolutionarily conserved sequences relative to background SNPs. For example, when using a phastCons score threshold of 0.4 to define conserved regions, approximately 28% of the rSNPs were retained compared to only 9% of the background SNPs. Significant differences in the frequency of SNPs that fall within conserved regions for the rSNP dataset and the background set were observed for all phastCons score thresholds above 0.1 (Figure 3).

Genomic regions close to the transcription start sites (TSS) of genes are generally more conserved between species than genomic regions far away from genes. Since research on regulatory genetic variation traditionally has been focused on the proximal promoter regions, and since the background SNPs were relatively evenly distributed in the region from 10 kb upstream of the TSS to the TSS, there was an overrepresentation of SNPs within the proximal promoter in the set of rSNPs compared with the background SNPs. To evaluate if this bias affected the enrichment of rSNPs in conserved regions relative the background, we compared the distributions of phastCons scores in the two datasets for SNPs located at different distances from the TSS. We binned all SNPs located from 10 kb to 2 kb upstream of the TSS, all SNPs located from 2 kb to 500 bases upstream of the TSS, all SNPs located from 500 bases upstream of the TSS to the TSS, and all SNPs from 100 bases upstream of the TSS to the TSS. The four bins contained 8, 20, 76, and 23 rSNPs, respectively. Due to the relatively low number of available rSNPs, separation of SNPs into smaller intervals was unpractical.

**Figure 4:**
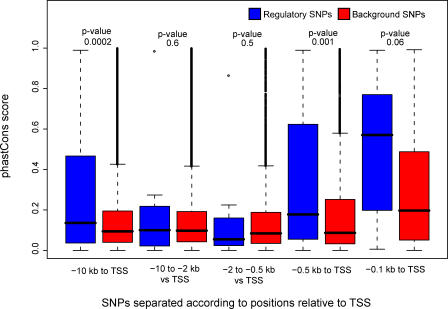
Distributions of Mean phastCons Scores for SNPs located at Different Distances from the TSS SNPs were given the mean phastCons scores from multiple alignments of human, chimp, mouse, rat, dog, chicken, fugu, and zebrafish in windows of 21 bp centered at the SNPs. For every interval a student's T-test was performed to check if there were significant differences in the distributions of phastCons values for the regulatory and background SNPs; the *p*-values from these tests are indicated above each pair of boxes.

Figure 4 shows that for SNPs located from 10 kb to 2 kb upstream, as well as 2 kb to 500 bases upstream of the TSS of the respective genes, there was no significant difference between the phastCons scores for the regulatory and background SNPs. However, for SNPs in the interval from 500 bp upstream to the TSS as well as for the full dataset, the phastCons score values were significantly higher for the regulatory than for the background SNPs (*p*-values 0.001 and 0.0002, respectively). Also in the interval closest to the TSS the phastCons score, values were higher for the regulatory than for the background SNPs, but the difference was not statistically significant. In the intervals closest to the TSS, the bias in location closer to the TSS for rSNPs is small or eliminated (in the interval from −500 bp to the TSS the median distances to the TSS were 168 bp and 237 bp for the regulatory and background SNPs respectively, and in the interval from 100 bp upstream to the TSS the median distance for both datasets was 51 bases). This suggests that the higher fraction of rSNPs in conserved regions relative to the background is not simply an effect of the rSNPs being located closer to the TSS than the background SNPs.

### TFBS Analysis Combined with Phylogenetic Footprinting Does Not Provide Additional Enrichment of Regulatory SNPs

**Figure 5:**
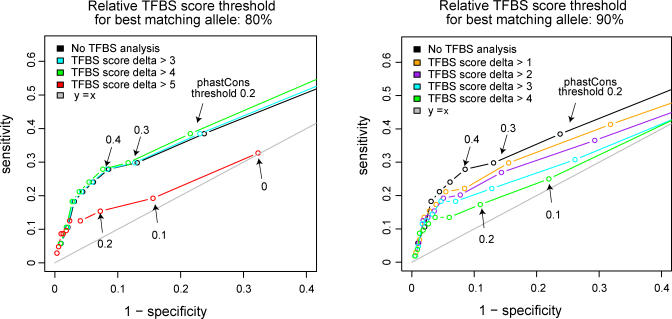
Combination of TFBS Analysis and Phylogenetic Footprinting Sensitivity of the predictions is plotted versus 1-specificity for phastCons score thresholds of 0, 0.1, 0.2, etc., up to 0.9. The whole range of values is only shown for the red curve; for the other curves, values for phastCons score thresholds 0 and 0.1 are outside the area covered by the plot. The curves correspond to different TFBS score delta thresholds. In the left panel, the relative TFBS score threshold for the best matching allele was 80%, in the right panel the relative TFBS score threshold for the best matching allele was 90%.

Although the TFBS analysis alone did not provide enrichment of rSNPs relative to the background, we tested if the intersection of TFBS analysis and phylogenetic footprinting could increase the enrichment given by phylogenetic footprinting alone. We counted the number of regulatory and background SNPs that both affected predicted TFBSs and were located in conserved regions (predicted rSNP ), for phastCons score thresholds 0.1 to 0.9 and for TFBS score delta thresholds between one and nine. We also counted the number of predicted rSNPs based on phylogenetic footprinting alone. Figure 5 shows the sensitivity (fraction of predicted rSNPs) versus 1—specificity (fraction of nonpredicted background SNPs) for the different thresholds, where the curves correspond to different TFBS score delta thresholds. When the relative TFBS score threshold for the best matching allele was set to 80% (the left panel), there was virtually no difference in performance between phylogenetic footprinting alone and when TFBS score delta thresholds of less than five was applied, and for larger score delta thresholds the sensitivity was very low. Also when the relative TFBS score threshold for the best matching allele is increased to 90% (the right panel), the application of TFBS analysis provides no enrichment compared with phylogenetic footprinting alone. Variations in particularly high scoring TFBS fail to disrupt sites, thus rSNP predictions for higher scoring TFBS candidates (Figure 5, right panel) are less predictive than those for predicted sites of lower initial scores (Figure 5, left panel).

### Availability of Additional Functional Information About the Gene Improves Regulatory SNP Discovery

Sometimes additional knowledge is available for the gene in which regulatory polymorphisms are searched, for example a researcher may know a set of transcription factors likely to be involved in the regulation of the gene in the context of the studied disease. When this type of information is available, it is possible to reduce the number of false positive TFBS predictions, since predictions of SNPs affecting the “wrong” transcription factor can be eliminated.

We analyzed a dataset of genes in which known TFBSs have been disrupted either by naturally occurring SNPs or by mutagenesis experiments. In this set, the transcription factors involved in the regulation of the corresponding genes had been determined experimentally. We searched a region from −3,000 bp to +500 bp relative to the TSS of every gene in the dataset for potential rSNPs affecting a binding site for the transcription factor associated with the gene. Table 1 shows that 12 of the 20 rSNPs with “prior knowledge” overlapped and affected a predicted binding site for the verified transcription factor, and in eight of these cases the rSNPs were located in genomic regions with a phastCons score above 0.4. Consistent with previous observations about the unusual predictive properties of an Sp1 binding profile [27], in two cases the Sp1 profile predicted decreased binding affinity toward a mutation in contrast to the published observation of increased affinity.

**Table 1:**
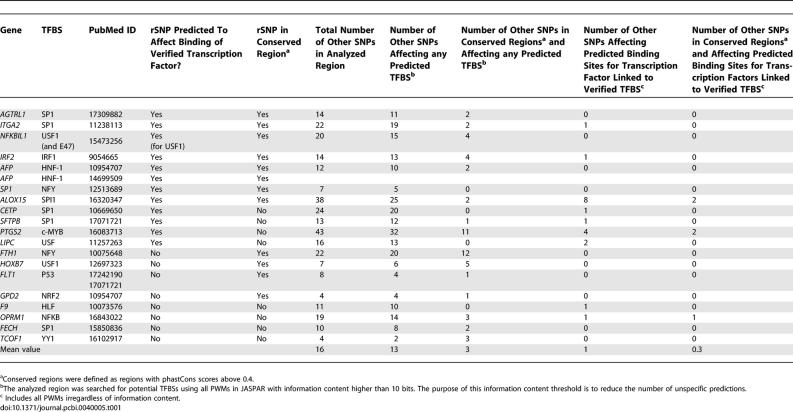
Analysis of Regulatory Mutations When the Affected Transcription Factor Binding Site Is Known

The analyzed regions (3,000 bp upstream to 500 bp downstream of the TSS of the analyzed genes) contained between four and 43 SNPs in addition to the analyzed rSNP. When the regions were analyzed with all PWMs in JASPAR with information content higher than 10 bits, between two and 32 (mean value 13) SNPs were predicted to affect a TFBS, and between zero and 11 of these were also located in conserved regions (mean value three). When the regions were analyzed with the PWM corresponding to the verified TFBS, the number of SNPs affecting predicted TFBS (in addition to the rSNP) was between zero and eight, with mean value one. Between zero and two of these were also located in conserved regions (mean value 0.3). This analysis indicates that when information regarding candidate transcription factors involved in the regulation of a gene is available, specificity is increased.

**Figure 6:**
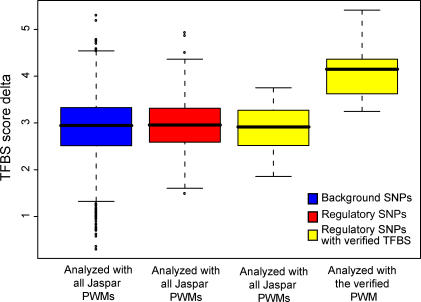
Distributions of TFBS Score Delta Values for Background SNPs, Regulatory SNPs, and Regulatory SNPs for Which the Affected TFBS Is Known In the three leftmost boxes the average score delta for all matches to any PWMs in the JASPAR database was collected for every SNP. In the rightmost box the score delta for the PWM corresponding to the verified PWM was collected for every SNP.

We also compared the score deltas obtained from analyzing the regulatory polymorphisms with known affected TFBS to the score deltas obtained when analyzing the larger data set used in Figure 2. When analyzing the regulatory polymorphisms with prior knowledge with all PWMs from the JASPAR database, the results were similar to those obtained for the larger set and the background. However, when the regulatory polymorphisms were analyzed with the PWMs for the respective verified TFBSs, the score delta was higher (Figure 6). In the analyses using all PWMs, we selected the mean score delta over all matches between the analyzed PWMs and the SNP, whereas in the analysis with the PWM of the verified transcription factor we used the PWM match giving the largest score delta for that particular PWM. This may have caused the lack of overlap between the interquartile ranges of score deltas in the “all” and “prior knowledge” analyses in Figure 6.

### The RAVEN Application

**Figure 7:**
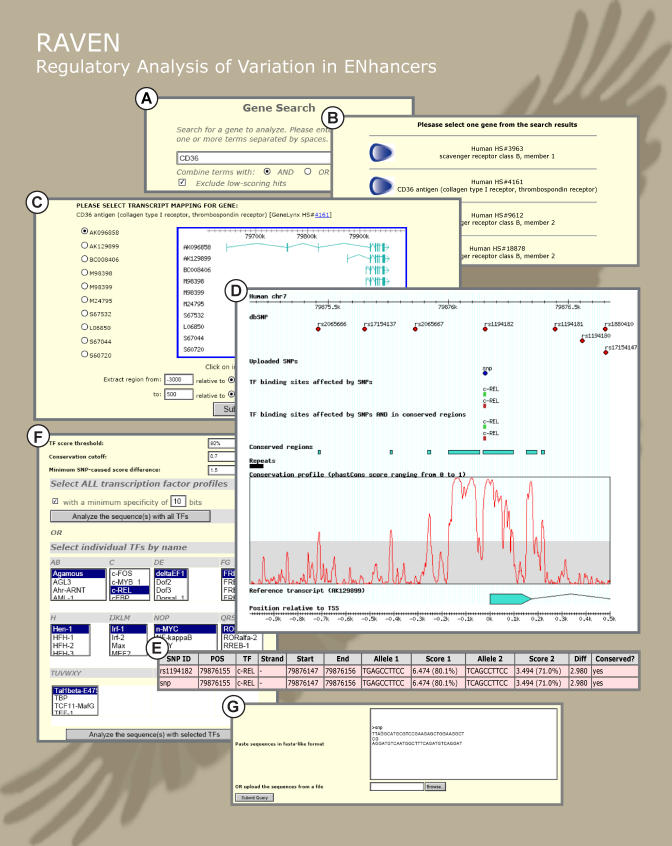
Overview of the RAVEN Web Interface (A) The search page. (B) The search results page where a list of genes corresponding to the search query is displayed. (C) The reference sequence selection page where the genomic location of the selected human sequence and cDNAs that map to it is displayed. (D) The graphical results view. (E) Table view of SNPs predicted to affect TFBSs. (F) Selection of TFBS profiles from the JASPAR database. (G) Upload of private SNP sequences.

To facilitate efficient analyses of the type presented in this paper, computational methods and newly implemented algorithms were developed as an integrated framework for rSNP analysis. The framework includes all the components for the location and extraction of data from genome and SNP databases, pattern detection, phylogenetic footprinting, and SNP effect estimation. To facilitate user access, we developed a flexible Web application that enables researchers to easily detect potential regulatory gene variation in their gene of interest (http://www.cisreg.ca). The organizational schema of the Web application is shown in Figure 7. The progression through analysis in RAVEN is event-driven, i.e., designed for a number of different application scenarios using different subcomponents of the system, and in different order. The components are:

#### Sequence selection and gene mapping.

Genes are located directly using keywords/identifiers (Figure 7A). The search engine is based on GeneLynx [33] and returns a list of human genes (Figure 7B). Upon selection of a gene from the list, the program displays the genomic location of the human sequence, with cDNAs that map to it (Figure 7C). The user selects the genomic coordinates for retrieval.

#### Result view is the central screen of the RAVEN application.

The result view contains a full control panel for changing analysis parameters and accessing other parts of the application. The results themselves can be viewed in one of two modes. (1) Graphical mode, in which all the submitted data and features detected by subsequent analysis are displayed as tracks in a genome browser-like view (Figure 7D). Genomic coordinates, SNPs from major SNP databases (currently dbSNP [34] and HGVbase [35]), conserved sequence segments, repeat sequences, the conservation profile, reference transcripts, and relative coordinates are displayed by default. The remaining tracks appear only after the corresponding data submission (user SNPs) or data selection (TFBS models) is made. (2) Table view, which appears when there are TFBSs in the window affected by SNPs, with a simple tabular list of SNPs affecting potential TFBS, positions of the SNPs and corresponding affected TFBSs, and the scores for the matches between both alleles of the SNP and the affected TFBS (Figure 7E). From the result view it is possible to switch to the other application components and to change the selections made previously.

#### Select transcription factor binding profiles.

To search for potential TFBSs affected by SNPs, the user can choose a subset of eukaryotic profiles from the JASPAR database [31] (Figure 7F). The algorithm used for transcription factor search and phylogenetic footprinting of the hits is described in [28].

#### Upload user SNPs.

Users can upload private sequence variation data to be analyzed for potential impact on TFBSs (Figure 7G). The uploaded SNPs are shown as separate tracks in Result View after uploading.

## Discussion

Identification of functional genetic variation associated with increased susceptibility to complex diseases is crucial for elucidating the biochemical mechanisms of disease and for pinpointing therapeutic targets [36]. Here we have introduced an in silico approach to the identification of candidate regulatory genetic variation in the human genome. A product of the research is the RAVEN system, which should be considered an exploratory tool for pinpointing candidate polymorphisms in transcription factor binding sites, facilitating further analyses of the polymorphism in vitro and in vivo.

Transcription factor binding profiles are appropriate for the study of genetic variation in binding sites. It was shown previously that scores obtained from PWM representations of TFBSs are proportional to the binding energy for the protein–DNA interaction [30]. This binding profile property makes the models suitable for ranking potential binding sites on two or more alleles. Providing additional support for the use of PWM binding profiles to predict altered TFBS is a recent study of binding affinities for the MAX A and MAX B transcription factors. Maerkl and Quake used a high-throughput microfluidic platform—capable of detecting low affinity transient binding events—to estimate the binding affinity between the two proteins and their respective target DNA sequences [37]. Their results show that MAX A and MAX B PWMs tend to overestimate differences in free binding energies between an optimal target sequence and sequence variants differing by three or more bp. However, most predictions for two bp deviations were correct, supporting the use of PWMs for assessing the impact of SNPs on consensus and near-consensus binding sites.

Due to the availability of data, a focus in the study of regulatory variation has been placed on the study of SNPs. The analysis of synthetic TFBSs with inserted mutations defines the expected ranges of score deltas between alleles for various mutation types. Interestingly, the median value of the score deltas for insertion and deletion mutations falls between the median value for one-base substitutions and that for two-base substitutions. This simulation indicates that, at the median, insertions and deletions are only slightly more disruptive of TFBSs than are one base substitutions, but perhaps importantly they have the capacity to generate more severe effects—as shown by the outliers in Figure 2. Therefore it may be appropriate, as new data sources emerge, to prioritize research on indels, as these sequence variations apparently have the potential to more severely alter a regulatory element.

Phylogenetic footprinting is useful for the selection of functional regulatory elements. The application of phylogenetic footprinting alone gives an enrichment of the experimentally verified regulatory variations versus the background SNPs, as shown in Figures 3 and 4. This is in agreement with previous reports suggesting that evolutionary conserved TFBS are more likely to be functional in vivo than are nonconserved sites [27]. Since nearly all SNPs both in the regulatory and background datasets affected a predicted binding site when no a priori selections of transcription factors were made, the combination of phylogenetic footprinting and TFBS analysis gave about the same enrichment of rSNPs as the phylogenetic footprinting alone, as shown in Figure 5.

A priori information about candidate transcription factors improves the identification of regulatory variations. The high number of SNPs results in a significant number of false predictions from our in silico method, even when TFBS analysis is combined with phylogenetic footprinting. To generate more meaningful predictions of TFBSs affected by polymorphisms, additional information relevant for the studied gene and in the clinical context must be incorporated. We have shown that when the transcription factor involved in the regulation of a particular gene is known, the number of SNPs that affect a potential TFBS in the region from 3,000 bases upstream to 500 bases downstream of the TSS is decreased from 13 to one. When also applying a conservation threshold (phastCons score above 0.4), the numbers are three and 0.3 (Table 1). This is encouraging and suggests that our in silico method can help pinpoint true rSNPs when additional information can guide the user in selecting candidate transcription factors. Suggestive prior data to motivate directed analysis for candidate binding sites for specific transcription factors can be derived from many sources. In addition to the scientific literature, candidate transcription factors can be selected based on associated Gene Ontology [38] terms in common with a target gene. High throughput proteomics initiatives such as the Human Protein Atlas program [39] can highlight transcription factors expressed in the tissues relevant for the target gene and the studied disease. Well designed expression profiling experiments can highlight genes likely to be regulated by a key transcription factor. For instance, Wang et al. focused their analysis on candidate rSNPs in putative binding sites for NFE2L2 based on genes observed (in microarray studies) to be induced by chemical compounds known to act via an NFE2L2-dependent mechanism [40]. Such prior knowledge is important in the search for regulatory polymorphisms.

In silico analysis of regulatory variation has been the focus of several recent efforts. Stepanova et al. compiled a collection of potential rSNPs in the upstream region of human genes [41]. The authors analyzed 72,824 SNPs in the regions from −2,000 bp to the transcription start site of 17,677 human genes using TFBS matrix models from the Transfac database [42], and identified SNPs affecting potential binding sites. Since Stepanova and colleagues used relative TFBS score delta thresholds, which are not generally comparable to our absolute score delta thresholds, we cannot directly compare the fraction of retained SNPs in the background SNP set in Figure 2 with their results. However, their fraction of retained SNPs (14,127 out of 72,824) does not contradict our results for the absolute TFBS score delta threshold of 5–6 (Figure 2). Zhao et al. presented a database called PromoLign that contains pre-computed SNP analyses of the 10 kb sequence upstream of more than 6,400 human–mouse orthologous gene pairs [43]. SNPs were assessed for overlap with potential TFBSs and with sequences conserved between human and mouse. RAVEN expands on the functions of PromoLign by enabling users to explore regulatory elements outside of the 5′ upstream proximal promoter, analyzing user-supplied SNPs and by using phastCons scores from multiple alignments for phylogenetic footprinting. One important feature of the RAVEN Web application is that the user is not restricted to regulatory polymorphisms around one annotated TSS, but can choose to study any region on the same chromosome as a defined mRNA sequence by selecting the appropriate start and end coordinates relative to the gene. Montgomery et al. introduced a predictive method for the detection of rSNPs based on a support vector machine trained on 22 properties of regions containing rSNPs [44]. Many properties were found to be poor predictors of rSNPs, including PWM scores. In fact, the distance from the transcription start site was found to be the property that gave the best enrichment of rSNPs. This observation, however, may be more reflective of an ascertainment bias as more researchers have studied candidate rSNPs in the immediate promoter regions. No online tool was provided for researcher use, although the underlying software is accessible online for advanced bioinformaticians.

### Conclusion

We have presented a computational method and a flexible, user-oriented Internet based tool for prediction of genetic variation with a potential to affect the regulation of the corresponding genes. In the absence of information about which transcription factor regulates a gene, our sequenced-based in silico analysis does not provide enough selectivity between polymorphisms with a documented impact on transcriptional regulation and background SNPs. However, if such prior information is available, we have demonstrated that our Web-based tool can aid experimental researchers in prioritizing which SNPs are most likely to affect the biological function of genomic regulatory elements.

## Methods

### Datasets.

*Synthetic SNP set.* Panels of simulated binding sites for transcription factors were generated from the distributions of bases at each position of all the TFBS frequency matrices available in the JASPAR database (123 matrices) [31]. A random base was inserted before and after the generated motif so that the sequences were *n*+2 bp long, where *n* is the length of the TFBS model. The insertion of extra bases before and after the motif enables the TFBS to shift one base in the mutated sequence if that would give a better match between the TFBS model and the sequence. Mutations of different types were introduced into the synthesized sequences: 1 bp substitutions, 2 bp substitutions at adjacent positions, two randomly placed 1 bp substitutions, 3 bp substitutions both in adjacent and at random positions, four randomly placed base pair substitutions, five randomly placed substitutions, one randomly placed 1 bp insertion, and one randomly placed 1 bp deletion. In case of insertion mutations, the random base after the site was removed so that the alleles are of same length and also of the same length as the other sequences of the corresponding site, i.e., *n*+2 bp. In case of deletion mutation, an extra random base was introduced in the end of the deleted allele so that both alleles were of length *n*+2 bp. Ten synthetic TFBS sequences were generated from each TFBS model (123 models), and ten mutants of each type were created for each generated wild-type sequence. This makes a total of 123 × 10 × 9 × 10 sequences = 98,400 sequences.

*Regulatory SNP set.* We searched the literature for variations with a documented regulatory role. All single base variations will be referred to as SNPs, although the frequency of the variations may be rare or unknown. The criteria for selecting SNPs to this set was that they should show allele-specific binding to nuclear extracts or transcription factors in vitro using electrophoretic mobility shift assays, and that the SNPs should show evidence of allele-specific transcription rates in reporter gene assays. We also used SNPs from previous collections of regulatory polymorphisms in human: 22 SNPs were taken from a collection created by Rockman and Wray [12], 17 were taken from the collection created by Buckland et al. [14], and 39 SNPs were extracted from the ORegAnno data base [13]. We did not require that the transcription factor affected by a particular SNP should be defined. rSNPs that were selected for in vitro studies based on the fact that they were located in evolutionarily conserved regions would have resulted in a bias in the phylogenetic footprinting analysis, and we have therefore not included such examples in our dataset. We limited the set to SNPs located in the regions from 10 kb upstream to the transcription start sites of human genes with available human–mouse orthologs (ortholog mapping was performed as in [45]). In total, 104 one-base substitution polymorphisms were collected. PubMed accession numbers and other details about the rSNP data set are shown in Dataset S1.

*Regulatory SNPs with known affected transcription factor binding site.* To generate a collection of regulatory polymorphisms for which the involved transcription factor has been experimentally determined, we mined the literature for examples of human TFBSs containing regulatory variation, either naturally occurring or generated in mutagenesis experiments. We applied the same inclusion criteria as for the rSNP set above, but only polymorphisms affecting binding sites for transcription factors for which there are PWMs available in JASPAR were included. The sequences for the regulatory variations with known affected TFBSs are provided in Dataset S2.

*Background SNP sets.* For background SNPs we downloaded from dbSNP those SNPs located in the upstream regions of human genes with a known mouse ortholog. We included SNPs in the regions stretching from −10 kb to the TSS (ortholog mappings and definitions of TSSs were determined as in [45]), unless a region is truncated by upstream genes in which case a smaller region was used. Only validated SNPs with minor allele frequency greater than 0.05 (in all populations) were included in the set. The dbSNP125 version of the database was used, in which SNP positions are calculated on the NCBI35 version of the human genome assembly. The total background SNP dataset used for the phylogenetic footprinting analysis contains 26,044 SNPs. For the analysis of overlap between SNPs and TFBSs, we used a subset of the total background dataset (4,000 SNPs), in which SNPs were selected randomly from the total background set. The background SNP datasets are available in Dataset S3 (26,044 SNPs) and Dataset S4 (4,000 SNPs). Defining the background SNPs as nonfunctional is a necessary simplification. As the background SNPs are taken from dbSNP without any functional analysis prior to inclusion, it is likely that the background dataset contains SNPs that have a regulatory function and hence the false positive predictions on the background set might include some true positive predictions. However, given the absence of a dataset with experimentally verified neutral SNPs in proximal promoter regions and the reasonable expectation that any true positives would be rare, the background SNP set is the only suitable dataset to which the rSNPs can be compared.

*Saturation mutagenesis data used in Figure S1.* Data was obtained from a saturation mutagenesis experiment of the heat shock element of the human HSP70.1 gene promoter [46]. The authors systematically mutated every base in the motif to all other base variants. A total of 55 sequences were tested covering 18 bases (i.e., 54 mutations plus wild-type). For every sequence, the relative free energy of the DNA–protein interaction (relative to the wild-type sequence) was measured by gel shift densitometry. From this analysis we compiled a list of sequence variants with corresponding relative free energies. We also used data from a saturation mutagenesis experiment in the Mnt repressor of the Salmonella phage P22 [30]. The authors mutated nine positions in the Mnt repressor and measured the relative free energy for every mutant sequence (relative to the wild-type sequence). From their analysis we again compiled a list of sequence variants with corresponding relative free binding energies. In the study of the Mnt repressor, the authors also presented a frequency matrix for the Mnt repressor that we used for scoring the sequences. The sequences used in the analysis of saturation mutagenesis experiments are shown in Dataset S5.

### Analysis.

*Analysis of synthetic mutated TFBS sequences.* The mutated and wild-type versions of the synthetic TFBS sequences were scored with the PWM used to generate a particular wild-type sequence, using perl scripts based on the TFBS framework [47]. The best possible match between the TFBS model (the model that was used to generate the wild-type sequence) and the DNA was collected for each sequence. A score delta was then calculated as the absolute value of the difference between the score delta for the wild-type sequence and the mutated sequence. To collect the score delta for a wild-type and mutated sequence pair we required that the relative score for the best matching allele should be at least 80% of the maximum score for the respective TFBS [24].

*TFBS analysis of documented regulatory SNPs and the background SNPs.* PWMs corresponding to all TFBSs in the JASPAR data base were used to score both alleles of the SNPs in the background and rSNP datasets. Scoring was performed as above, but with two modifications. (1) We required a predicted binding site to be located at the same position in the sequence for both alleles of a SNP. This is the same methodology as is used in the Web interface to our application. (2) If several matches were obtained between a SNP and a particular TFBS model (fulfilling the requirement that the highest-scoring allele should give a score of at least 80% of the of the maximum score for the respective TFBS), we collected the hit that gave the largest score delta between the sequence variants since we wanted to calculate the fraction of retained SNPs for different TFBS score deltas. Sometimes a SNP overlapped potential binding sites for more than one TFBS model. To calculate the fraction of retained SNPs for various TFBS score delta thresholds we collected, for each SNP, the maximum score delta over all TFBS models (rather than the score delta for the TFBS model that gave the largest absolute score for the best matching allele). We measured the fraction of retained SNPs in respective datasets for the absolute TFBS score delta thresholds in one-unit increments from one to nine.

*Phylogenetic footprinting analysis of regulatory SNPs and the background SNPs.* We used the phastCons scores from multiple alignments of eight vertebrate genomes available in the UCSC genome browser (the May 2004 release) to perform phylogenetic footprinting. SNPs were assigned the mean phastCons score in a window of 21 bases (ten bases upstream and downstream of the SNP). Several other window sizes were tested, from one to 41 bp, but the 21 bp window gave the best enrichment of rSNPs.

*Analysis of regulatory SNPs with known affected TFBS.* The examples of regulatory variation from the literature for which the affected TFBS are known were analyzed using the Web interface to our application (RAVEN, see description below). The following parameters were used for the analysis: the phastCons threshold for sequence conservation between species was 0.4. The threshold for a match between the top-scoring allele of a variation and a TFBS model was 80%. The absolute score difference between the two alleles of a variation was required to be greater than 1.5 [24]. The TFBS analysis of the different SNP datasets shown in Figure 6 were performed as above, but with the exception that the average score delta from all PWMs that fulfilled the search criteria for a particular SNP was collected instead of collecting only the PWM that gave the largest score delta.

*Correlation analysis of relative free energy of mutated binding sites versus TFBS score delta for Figure S1.* The mutated and wild-type sequences in the saturation mutagenesis experiments were scored with the PWMs corresponding to position frequency matrices for the respective transcription factors [24]. A score delta was computed for every sequence variant by subtracting the absolute score for the wild-type sequence from the score for the mutated sequence. For each sequence the optimal match between the TFBS model and the analyzed sequence was used, with the requirement that the motif should overlap the mutated base. The score deltas for all the sequence variants were then correlated to the experimental relative free binding energies.

The position frequency matrix for the heat shock element was taken from Transfac [42]. The matrix has Transfac accession number M00146 and is defined by:

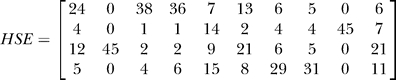
For the analysis of mutated versions of the Salmonella phage P22 Mnt repressor, we used a published position frequency matrix [30]. The matrix is defined by:

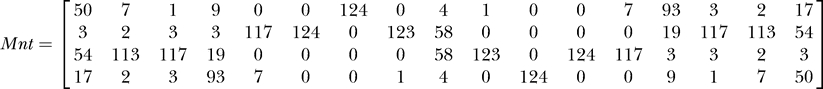



The frequency matrices were transformed into weight matrixes using the TFBS bioinformatics programming framework [47]. Functions from the same framework were also used for searching sequences for putative binding sites. Analyses of the correlation of TFBS score deltas and relative free binding energy for the sequence variants were performed in R [48].

### The RAVEN Web application.

The application is implemented in object-oriented Perl (Conway, 1999) with extensive use of Bioperl [49] and TFBS [47] bioinformatics programming frameworks. The system consists of several database components, an application engine layer, and a Web interface layer. It is deployed on a dual-CPU Linux/i686 server.

*Data.* The dbSNP [34] sequence data was downloaded from NCBI ftp site (ftp://ftp.ncbi.nlm.nih.gov/). Genomic sequence data with the positions of repeat sequences, mappings of cDNAs and dbSNP SNPs to the genome, as well as phastCons scores, were obtained from the UCSC Genome Informatics repository (http://genome.ucsc.edu/) [50]. HGVbase (http://www.hgvbase.org/) [35] data was obtained directly from the master database at Karolinska Institutet.

## Supporting Information

Figure S1 Correlation between Relative Free Energies and TFBS Score Deltas of Mutated Binding Sites Two published saturation mutagenesis experiments demonstrate, as previously shown by Stormo and colleagues [30], how PWM representations of TFBSs can be used to estimate the effect of genetic variation in a regulatory region on the binding affinity of the corresponding transcription factor. Positions in the heat shock element in the human *HSP70.1* gene [46] and in the Mnt repressor of the Salmonella phage P22 [30] were systematically mutated, followed by in vitro determination of the relative free energy of binding between the proteins and the sequence variants. The sequence variants were here scored with the PWM for the corresponding transcription factors, and score deltas (i.e., the difference in assigned scores by the PWM) between the wild-type allele and the different mutated alleles were collected. The TFBS score deltas for a) HSE and b) Mnt repressor are plotted against the published differences in relative free energy. In both examples, the relative free energy is significantly correlated with the score delta (r^2^ = 0.73, *p*-value = 2.2e−16 for HSE, and r^2^ = 0.61, *p*-value = 7.571e−07 for Mnt).
(296 KB PDF).

Dataset S1 rSNPs (18 KB TDS).

Dataset S2 Prior Knowledge rSNPs (30 KB DOC).

Dataset S3 The 26,044 Background SNPs (3.8 MB TDS).

Dataset S4 The 4,000 Background SNPs (5.8 MB TDS).

Dataset S5 Saturation Mutagenesis Data (5.95 KB TDS).

## Abbreviations

PWM: position-specific weight matrices
RAVEN: regulatory analysis of variation in enhancers
rSNP: regulatory SNP
SNP: single nucleotide polymorphism
TFBS: transcription factor binding site
TSS: transcription start site

## References

[pcbi-0040005-b001] Kuriki C, Tanaka T, Fukui Y, Sato O, Motojima K (2002). Structural and functional analysis of a new upstream promoter of the human FAT/CD36 gene. Biol Pharm Bull.

[pcbi-0040005-b002] Zabetian CP, Anderson GM, Buxbaum SG, Elston RC, Ichinose H (2001). A quantitative-trait analysis of human plasma-dopamine beta-hydroxylase activity: evidence for a major functional polymorphism at the DBH locus. Am J Hum Genet.

[pcbi-0040005-b003] Rigat B, Hubert C, Alhenc-Gelas F, Cambien F, Corvol P (1990). An insertion/deletion polymorphism in the angiotensin I-converting enzyme gene accounting for half the variance of serum enzyme levels. J Clin Invest.

[pcbi-0040005-b004] Bosma PJ, Chowdhury JR, Bakker C, Gantla S, de Boer A (1995). The genetic basis of the reduced expression of bilirubin UDP-glucuronosyltransferase 1 in Gilbert's syndrome. N Engl J Med.

[pcbi-0040005-b005] De Gobbi M, Viprakasit V, Hughes JR, Fisher C, Buckle VJ (2006). A regulatory SNP causes a human genetic disease by creating a new transcriptional promoter. Science.

[pcbi-0040005-b006] Alj Y, Georgiakaki M, Savouret JF, Mal F, Attali P (2004). Hereditary persistence of alpha-fetoprotein is due to both proximal and distal hepatocyte nuclear factor-1 site mutations. Gastroenterology.

[pcbi-0040005-b007] Dawson S, Wiman B, Hamsten A, Green F, Humphries S (1993). The two allele sequences of a common polymorphism in the promoter of the plasminogen activator inhibitor-1 (PAI-1) gene respond differently to interleukin-1 in HepG2 cells. J Biol Chem.

[pcbi-0040005-b008] Grant PJ, Humphries SE (1999). Genetic determinants of arterial thrombosis. Baillieres Best Pract Res Clin Haematol.

[pcbi-0040005-b009] Jormsjö S, Ye S, Moritz J, Walter DH, Dimmeler S (2000). Allele-specific regulation of matrix metalloproteinase-12 gene activity is associated with coronary artery luminal dimensions in diabetic patients with manifest coronary artery disease. Circ Res.

[pcbi-0040005-b010] Bai F, Rankinen T, Charbonneau C, Belsham DD, Rao DC (2004). Functional dimorphism of two hAgRP promoter SNPs in linkage disequilibrium. J Med Genet.

[pcbi-0040005-b011] Argyropoulos G, Rankinen T, Bai F, Rice T, Province MA (2003). The agouti-related protein and body fatness in humans. Int J Obes Relat Metab Disord.

[pcbi-0040005-b012] Rockman MV, Wray GA (2002). Abundant raw material for cis-regulatory evolution in humans. Mol Biol Evol.

[pcbi-0040005-b013] Montgomery SB, Griffith OL, Sleumer MC, Bergman CM, Bilenky M (2006). ORegAnno: an open access database and curation system for literature-derived promoters, transcription factor binding sites and regulatory variation. Bioinformatics.

[pcbi-0040005-b014] Buckland PR, Hoogendoorn B, Coleman SL, Guy CA, Smith SK (2005). Strong bias in the location of functional promoter polymorphisms. Hum Mutat.

[pcbi-0040005-b015] Pastinen T, Hudson TJ (2004). Cis-acting regulatory variation in the human genome. Science.

[pcbi-0040005-b016] Cavalieri D, Townsend JP, Hartl DL (2000). Manifold anomalies in gene expression in a vineyard isolate of Saccharomyces cerevisiae revealed by DNA microarray analysis. Proc Natl Acad Sci U S A.

[pcbi-0040005-b017] Jin W, Riley RM, Wolfinger RD, White KP, Passador-Gurgel G (2001). The contributions of sex, genotype and age to transcriptional variance in Drosophila melanogaster. Nat Genet.

[pcbi-0040005-b018] Morley M, Molony CM, Weber TM, Devlin JL, Ewens KG (2004). Genetic analysis of genome-wide variation in human gene expression. Nature.

[pcbi-0040005-b019] Cowles CR, Hirschhorn JN, Altshuler D, Lander ES (2002). Detection of regulatory variation in mouse genes. Nat Genet.

[pcbi-0040005-b020] Knight JC, Keating BJ, Rockett KA, Kwiatkowski DP (2003). In vivo characterization of regulatory polymorphisms by allele-specific quantification of RNA polymerase loading. Nat Genet.

[pcbi-0040005-b021] Pastinen T, Sladek R, Gurd S, Sammak A, Ge B (2004). A survey of genetic and epigenetic variation affecting human gene expression. Physiol Genomics.

[pcbi-0040005-b022] Ronald J, Brem RB, Whittle J, Kruglyak L (2005). Local regulatory variation in Saccharomyces cerevisiae. PLoS Genet.

[pcbi-0040005-b023] GuhaThakurta D, Xie T, Anand M, Edwards SW, Li G (2006). Cis-regulatory variations: a study of SNPs around genes showing cis-linkage in segregating mouse populations. BMC Genomics.

[pcbi-0040005-b024] Stormo GD (2000). DNA binding sites: representation and discovery. Bioinformatics.

[pcbi-0040005-b025] Tronche F, Ringeisen F, Blumenfeld M, Yaniv M, Pontoglio M (1997). Analysis of the distribution of binding sites for a tissue-specific transcription factor in the vertebrate genome. J Mol Biol.

[pcbi-0040005-b026] Fickett JW (1996). Quantitative discrimination of MEF2 sites. Mol Cell Biol.

[pcbi-0040005-b027] Wasserman WW, Palumbo M, Thompson W, Fickett JW, Lawrence CE (2000). Human-mouse genome comparisons to locate regulatory sites. Nat Genet.

[pcbi-0040005-b028] Lenhard B, Sandelin A, Mendoza L, Engstrom P, Jareborg N (2003). Identification of conserved regulatory elements by comparative genome analysis. J Biol.

[pcbi-0040005-b029] Wasserman WW, Sandelin A (2004). Applied bioinformatics for the identification of regulatory elements. Nat Rev Genet.

[pcbi-0040005-b030] Fields DS, He Y, Al-Uzri AY, Stormo GD (1997). Quantitative specificity of the Mnt repressor. J Mol Biol.

[pcbi-0040005-b031] Sandelin A, Alkema W, Engstrom P, Wasserman WW, Lenhard B (2004). JASPAR: an open-access database for eukaryotic transcription factor binding profiles. Nucleic Acids Res.

[pcbi-0040005-b032] Siepel A, Bejerano G, Pedersen JS, Hinrichs AS, Hou M (2005). Evolutionarily conserved elements in vertebrate, insect, worm, and yeast genomes. Genome Res.

[pcbi-0040005-b033] Lenhard B, Hayes WS, Wasserman WW (2001). GeneLynx: a gene-centric portal to the human genome. Genome Res.

[pcbi-0040005-b034] Sherry ST, Ward MH, Kholodov M, Baker J, Phan L (2001). dbSNP: the NCBI database of genetic variation. Nucleic Acids Res.

[pcbi-0040005-b035] Fredman D, Munns G, Rios D, Sjoholm F, Siegfried M (2004). HGVbase: a curated resource describing human DNA variation and phenotype relationships. Nucleic Acids Res.

[pcbi-0040005-b036] Hudson TJ (2003). Wanted: regulatory SNPs. Nat Genet.

[pcbi-0040005-b037] Maerkl SJ, Quake SR (2007). A systems approach to measuring the binding energy landscapes of transcription factors. Science.

[pcbi-0040005-b038] Ashburner M, Ball CA, Blake JA, Botstein D, Butler H (2000). Gene ontology: tool for the unification of biology. The Gene Ontology Consortium. Nat Genet.

[pcbi-0040005-b039] Uhlen M, Bjorling E, Agaton C, Szigyarto CA, Amini B (2005). A human protein atlas for normal and cancer tissues based on antibody proteomics. Mol Cell Proteomics.

[pcbi-0040005-b040] Wang X, Tomso DJ, Chorley BN, Cho HY, Cheung VG (2007). Identification of polymorphic antioxidant response elements in the human genome. Hum Mol Genet.

[pcbi-0040005-b041] Stepanova M, Tiazhelova T, Skoblov M, Baranova A (2006). Potential regulatory SNPs in promoters of human genes: a systematic approach. Mol Cell Probes.

[pcbi-0040005-b042] Matys V, Fricke E, Geffers R, Gossling E, Haubrock M (2003). TRANSFAC: transcriptional regulation, from patterns to profiles. Nucleic Acids Res.

[pcbi-0040005-b043] Zhao T, Chang LW, McLeod HL, Stormo GD (2004). PromoLign: a database for upstream region analysis and SNPs. Hum Mutat.

[pcbi-0040005-b044] Montgomery SB, Griffith OL, Schuetz JM, Brooks-Wilson A, Jones SJ (2007). A survey of genomic properties for the detection of regulatory polymorphisms. PLoS Comput Biol.

[pcbi-0040005-b045] Ho Sui SJ, Mortimer JR, Arenillas DJ, Brumm J, Walsh CJ (2005). oPOSSUM: identification of over-represented transcription factor binding sites in co-expressed genes. Nucleic Acids Res.

[pcbi-0040005-b046] Cunniff NF, Morgan WD (1993). Analysis of heat shock element recognition by saturation mutagenesis of the human HSP70.1 gene promoter. J Biol Chem.

[pcbi-0040005-b047] Lenhard B, Wasserman WW (2002). TFBS: computational framework for transcription factor binding site analysis. Bioinformatics.

[pcbi-0040005-b048] Team RDC (2005). R: A language and environment for statistical computing.

[pcbi-0040005-b049] Stajich JE, Block D, Boulez K, Brenner SE, Chervitz SA (2002). The Bioperl toolkit: Perl modules for the life sciences. Genome Res.

[pcbi-0040005-b050] Karolchik D, Baertsch R, Diekhans M, Furey TS, Hinrichs A (2003). The UCSC Genome Browser Database. Nucleic Acids Res.

