# Supplementary material for: In Silico Detection of Sequence Variations Modifying Transcriptional Regulation
Source: PLoS Comput Biol. 2008 Jan 18;4(1):e5. doi: 10.1371/journal.pcbi.0040005 (PMC2211530; doi:10.1371/journal.pcbi.0040005)
Supplement: Supplementary file 1 [file pcbi.0040005.sg001.pdf]

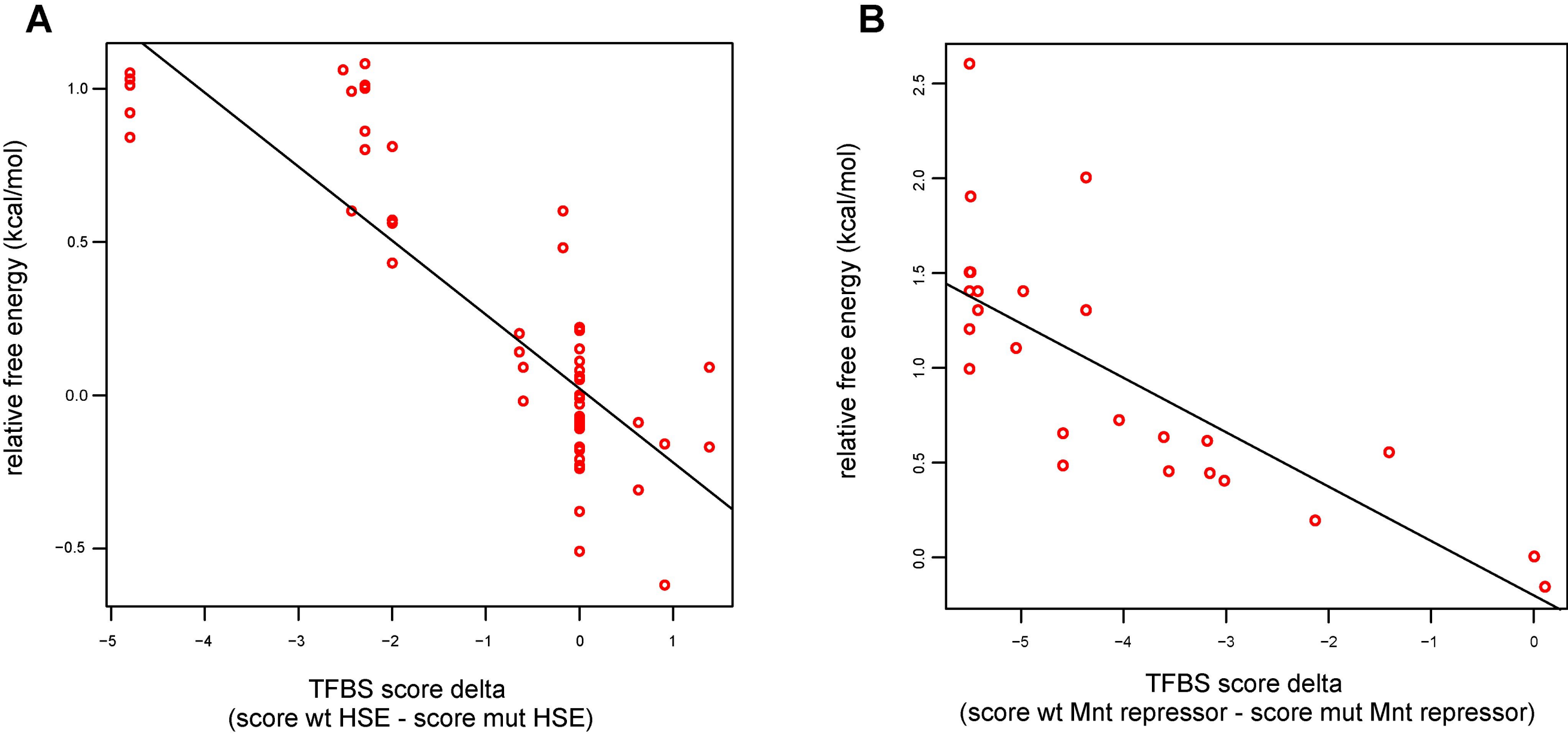

A
B
relative free energy (kcal/mol)
1.0
0.5
0.0
−0.5
−5
−4
−3
−2
−1
0
1
TFBS score delta
(score wt HSE - score mut HSE)
2.5
2.0
1.5
1.0
0.5
0.0
relative free energy (kcal/mol)
−5
−4
−3
−2
−1
0
TFBS score delta
(score wt Mnt repressor - score mut Mnt repressor)
