# Supplementary material for: In Silico Detection of Sequence Variations Modifying Transcriptional Regulation
Source: PLoS Comput Biol. 2008 Jan 18;4(1):e5. doi: 10.1371/journal.pcbi.0040005 (PMC2211530; doi:10.1371/journal.pcbi.0040005)
Supplement: Supplementary file 2 [file pcbi.0040005.sd002.doc]

SNPs are presented in "fasta like format" that can be uploaded to the RAVEN web application. The data corresponds to the SNPs in Table 1, with annotations "gene_TF".

>AGTRL1_SP1

GTGCTCTCCGCCCTCCTGTTCTCACCCCCT

T|C

CCATCCAATCTAAATGGGACACATTATGCA

>ITGA2_SP1

CCGGTGTTTGCGGAATCAGGAGGGGCGGGC

T|C

GGGGCGGGCCCTCGGCGCTGCAGGAGCTGC

>NFKBIL1_USF1

GGCTGGAGGAAATGGCGCAAGCAGAGACGC

A|T

GGTGGAGGACGGAAGTGAACTGTGAGGGGC

>IRF2_IRF1

CCCGGCTTGCCTGAGAACAGTCCAGATCGA

A|C

AGCAAAACAAGGAGAGGAAACTGCGAAGAG

>AFP_HNF-1

TAAAGTAACAGATATACCAACAAAAGGTTA

C|A

TAGTTAACAGGCATTGCCTGAAAAGAGTAT

>AFP_HNF-1

TACAAATAACCGCTATGCTGTTAATTATTG

G|A

CAAATGTCCCATTTTCAACCTAAGGAAATA

>SP1_NFY

CTGCAAGTCCCTCCTTTCCCCCTCCCTCAT

T|G

GGGCGGGGCAGCAGAGAAGGGGCGGGGCCT

>ALOX15_SPI1

CTCGCCTGCCTGCTGTACCAGGCGTTGATT

C|T

CCTCTTCCTGCGTGTTTTCGGTCCAAATCC

>CETP_SP1

GCAGCCAATGATCTCAGAGGCTGTATACCC

C|A

CCCAGAGTTATTTTATGCATATCAAGGAAA

>SFTPB_SP-1

GCACCTCTGCAGCCTGGGTACCCTGCTTGG

T|C

GCATGGCCCCTTATAGCTGGGCGGGGCGTG

>PTGS2_c-MYB

ATTAATTAGATGGAAGGGAGATTTTGACAG

T|C

TGGAATTTCATCTTTGCTTTTGTTTAACAT

>LIPC_USF

TCTTTGCTTCTTCGTCAGCTCCTTTTGACA

C|T

GGGGGTGAAGGGTTTTCTGCACCACACTTT

>FTH1_NFY

TCAGGCCCGCCCCGGCCAATCAGCGCCGCC

CA

GCCCCGAGCCCGCTCCTTCCGGTGGGCGCG

>HOXB7_USF1

ACCCCCTTTTTCCTCTCCGGACCACGTGAC

C|G

CCGCCCACGTGACGTCCCCTCCGCCAATGG

>FLT1_P53

ACTGCGAACCAGCTCAGGTCCCAGGGGAGC

G|A

TGTCCGTGTCTTTTTCGGCCGCATTGATGC

>GPD2_NRF2

AGGGAGCAGGCGCGGGAGCGGGAGGAGGAA

G|T

TCGGGAAGAGGGAAGGCGGGGGCGCGGCCC

>F9_HLF

CCACTATGCCTATCTCCATTCTGAAGATGT

G|T

TCACTTCCTGTTTCAGACTCAAATCAGCCA

>OPRM1_NFKB

AATTGGCAGTAGGGATGGAAGAGCATTGGG

G|A

TTTTAGGGCTGTTAGGGTTTCATCAAGCCA

>FECH_SP1

CCCTGCGTGAAAATGGGCCCGGGGGCTGAG

C|G

TAGAAGCCCGCTCCCCACCACCATCGCAGG

>TCOf1_YY1

GGGAGTTCAGAGATGAGTAAAACGCAGACC

C|T

AGCCATCAGAGAATTAACAAGGAAGATAAA
